# Historical copper mining contamination assessed using dendrochemical analysis in Southeastern Sweden

**DOI:** 10.1007/s11356-025-36721-9

**Published:** 2025-07-12

**Authors:** Jonatan F. Uusitalo, Hans W. Linderholm, Björn E. Gunnarson

**Affiliations:** 1 Department of Physical Geography, Stockholm University, Stockholm, Sweden; 2 Department of Earth Sciences, University of Gothenburg, Gothenburg, Sweden

**Keywords:** Dendrochemistry, Dendroforensic, Pollution, ED-XRF, Tree-rings, Environmental monitoring

## Abstract

Heavy metal pollution from untreated or poorly managed mining waste is a major environmental concern, leading to the leaching of contaminants into surrounding ecosystems. Traditional monitoring methods are costly and limited in their ability to reconstruct historical contamination trends. Dendrochemical methods offer a promising alternative for assessing long-term pollution dynamics. This study investigates temporal patterns of copper (Cu), nickel (Ni), and zinc (Zn) accumulation in tree rings from 22 European aspens (*Populus tremula*) growing near an abandoned copper mining field in southeastern Sweden. Tree rings were analyzed using energy-dispersive X-ray fluorescence (ED-XRF), while corresponding soil samples were examined for heavy metal concentrations and pH. Considerable heterogeneity in Cu and pH was observed, with elevated Cu levels across the site. Although no significant correlations were found between soil and tree-ring metal concentrations, increased accumulation of Cu, Ni, and Zn was detected in the trees. Temperature showed a more consistent influence on metal uptake than precipitation. Anomalous uptake increased under warmer and wetter conditions but was absent during severe drought. This study emphasizes the potential of dendrochemical methods for monitoring historical pollution. It highlights the need to refine sampling strategies to better account for localized soil and climatic factors, thereby improving the reliability of dendrochemical methods in assessing pollution trends.

## Introduction

Since the onset of the Industrial Revolution, the expansion of human civilization and ever-increasing economic growth have accelerated the anthropogenic impact on ecological systems. While heavy metals occur naturally in soils through pedogenic processes, industrialization and urbanization have significantly increased their circulation in air, water, and soils to levels of global concern (Timothy and Tagui Williams 2019). The mining industry alone produces billions of tons of toxic waste annually, with numerous sites left untreated or improperly managed, resulting in acid mine drainage (AMD) and the release of heavy metals into the surrounding environment (Akcil and Koldas 2006). As the “green” energy revolution relies on non-renewable raw materials, mining activity and waste production are projected to increase (Herrington 2021). This trend coincide with climate change, which involves shifts in temperature and precipitation patterns, as well as increase in the frequency and intensity of extreme weather events (Almazroui et al. 2021). Climatic variations may enhance metal mobility and contamination from legacy mining sites. Elevated temperatures accelerate sulfide oxidation in mine waste (Biswas et al. 2018), while intensified freeze–thaw cycles can aggravate erosion and runoff, mobilizing contaminants during spring floods and thaw-related events (Punia 2021). Altered precipitation patterns and more frequent extreme weather events may disperse contaminants over larger areas, resulting in severe environmental consequences. These developments highlight the importance of understanding how past contamination responded to climatic variations to develop effective mitigation strategies, especially as mining waste production increases. However, conventional environmental monitoring methods are limited by high costs, time demands, and the need for long-term site-specific data collection to reconstruct historical conditions (Morrison 2000a, 2000b). To address these challenges, dendrochemistry, a sub-discipline of dendrochronology, has emerged as a promising tool for environmental monitoring. By analyzing the chemical composition of annual tree rings, dendrochemistry provides high-resolution proxy data that can be used to reconstruct long-term environmental change (Balouet 2015), offering temporal insights that traditional methods cannot provide. Although a relatively young field requiring further research (Canning et al. 2023), dendrochemistry has been successfully applied to reconstruct pollution from various sources, including car traffic (Locosselli et al. 2019), tourism (Li et al. 2023), smelters (Kirdyanov et al. 2020), mining sites (Märten et al. 2015), and industrial activity (Cui et al. 2013; Rocha et al. 2020). In recent decades, significant technical advances have been made, including energy-dispersive X-ray fluorescence (ED-XRF), a non-destructive, high-resolution method capable of simultaneously analyzing multiple elements, particularly first-row transition metals (Binda et al. 2021). However, despite technical improvements, dendrochemical research remains limited in scope, with many studies relying on small sample sizes, typically fewer than ten cores due to cost and time constraints (Balouet and Oudijk 2005; Balouet et al. 2012; Canning et al. 2023; Garbe-Schönberg et al. 1997; Hevia et al. 2018; Märten et al. 2015; Zyskowski et al. 2021). Moreover, there is a notable lack of published studies in Nordic regions (Canning et al. 2023). In Sweden, numerous closed mines and contaminated sites continue to leach heavy metals into the environment, posing risks to local ecosystems (Länsstyrelsen I Västerbotten 2012).

This study investigates whether temporal patterns of heavy metal contamination in soil are reflected in tree rings from European aspen (*Populus tremula*). To address this, we will analyze a comparatively large dendrochemical dataset of 22 trees and associated soil samples from an abandoned copper mining field in southeastern Sweden using the ED-XRF technique. Specifically, we will (i) examine whether tree rings reflect the levels of specific elements and pH in the soil, (ii) investigate the impact of temperature and precipitation on the absorption of contaminants, and (iii) establish if dendrochemical signals are consistent across multiple trees. Based on these findings, we propose refinements to sampling techniques that could enhance the reliability of dendrochemical data for future environmental monitoring.

## Method

### Study site

The Närstad copper mining field (58°12′36′′N 15°54′32′′E) is located approximately 5 km west-northwest of Åtvidaberg in Östergötland County, Sweden, and has a documented mining history dating back to the fourteenth century. Short-lived mining attempts occurred between the mid-1500 s and early 1600 s, after which the area remained dormant until the early eighteenth century. Mining resumed in 1730 and reached peak production in 1869. Following the depletion of ore deposits, operations ceased, and the associated Åtvidaberg copper smelter was decommissioned in 1902 (Andersson n.d.), marking the end of industrial-scale copper production. The mining field is now characterized by abandoned mine pits and extensive waste rock piles, contributing to local heavy metal contamination. Copper (Cu) is the primary pollutant, with zinc (Zn) and nickel (Ni) as secondary contaminants (Wrafter 2004). This study focuses on three mines within the mining field: Mormorgruvan, Haggruvan, and Varpgruvan, as well as a main drainage ditch and a reference site 3 km to the south.

The mines are situated on elevated terrain surrounded by mixed coniferous and deciduous forest. Soil types include post-glacial clay, peat, and sandy till. The soils have been substantially altered near the waste rock piles, resulting in shallow and poorly developed topsoil layers.

#### Mormorgruvan

**Fig. 1:**
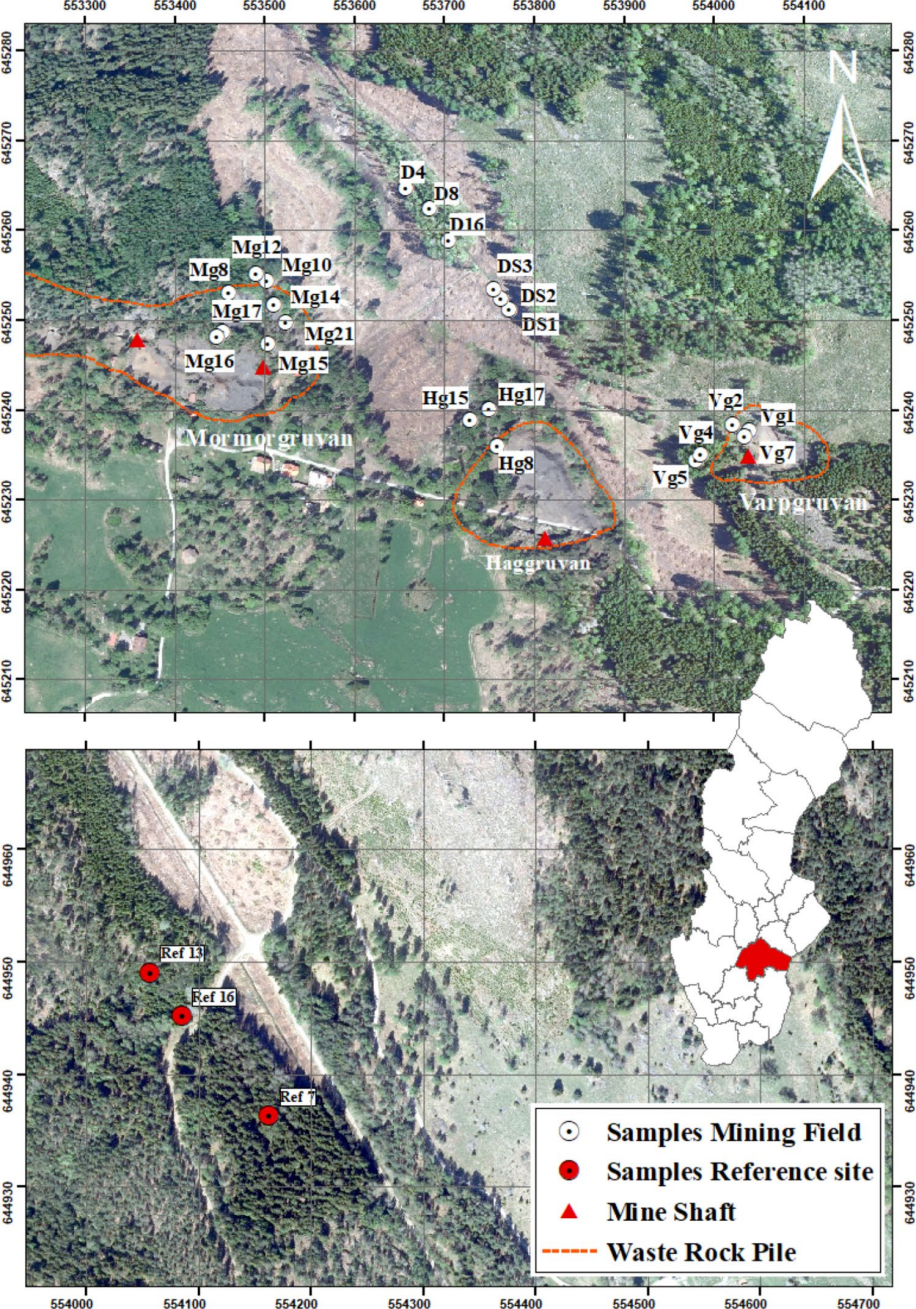
Overview of sampling location at Närstad mining field (top image) and uncontaminated reference site (bottom image). Lake Fyrsjön is located north, outside the figure. Samples labeled DS1-DS3 represent soil samples taken from the drainage ditch. The grid size in the map is 100 × 100 m. Coordinate system: Sweref 99TM

As the largest mine in the study area, Mormorgruvan reopened in 1791 and remained active until 1872, reaching a final depth of 407 m. The surrounding landscape is varied, consisting of mixed forest, drainage channels, and heterogeneous soils ranging from postglacial clay to coarse waste rock. Its main waste rock pile covers approximately 25,000 m^2^ and is estimated to contain between 40,000 and 80,000 m^3^ of material, with a height of 8 to 10 m and sulfide content ranging from 10 to 20%. Two smaller piles are situated east of the mine shaft, separated by patches of woodland (Fig. 1). East of Mormorgruvan lies the Nygruvan pit, which also receives runoff from the main pile. The surrounding soil is predominantly waste rock with minimal topsoil.

#### Haggruvan

Approximately 300 m east of Mormorgruvan lies Haggruvan, which was mined intermittently between 1772 and 1818, with renewed activity from 1826 to 1828. Minor operations continued sporadically until final closure in 1868 at a depth of 207 m. Its waste rock pile spans roughly 10,000 m^2^, with an estimated volume of 20,000 to 40,000 m^3^ and heights ranging from 2 to 8 m. (The sulfide content of this pile is currently unknown.) To the west and north, a crescent-shaped stand of oak and aspen forest borders the site. Glacial clay and sandy moraine dominate the local soil composition.

#### Varpgruvan

Situated in the eastern section of the Närstad mining field, Varpgruvan operated between 1861 and 1879 and contains the smallest waste rock pile among the three mines. This pile covers an area of approximately 4000 m^2^, with a volume estimated between 6000 and 10,000 m^3^ and a height of 2 to 3 m. To the west, the site is bordered by forest. Notably, this pile exhibits the highest sulfide content of all examined sites, ranging from 40 to 60%.

#### Main drainage ditch

An 800-m-long drainage ditch connects Varpgruvan to Lake Fyrsjön, collecting surface runoff from all three mine sites. About 200 m downstream, a forested area dominated by European aspen is situated, underlain primarily by peat.

#### Reference site

Located 3 km south of the mining field, the reference site is dominated by coniferous trees, with oak and birch as the main deciduous species. It lies in a low-lying area bisected by a gravel road. Evidence of forest thinning is visible. The soil consists primarily of podzols interspersed with coarse stones and boulders.

### Tree core collection and preparation

In September 2022, two radii from each of a total of 87 living European aspen trees, which showed no visible scarring or damage, were extracted using a 10 mm increment corer at breast height (1.3 m). The trees located at Mormorgruvan (Mg), Haggruvan (Hg), Varpgruvan (Vg), the drainage ditch (D), and the unpolluted reference site (Ref) were labeled sequentially with a number indicating the tree’s sampling order at each location. The samples were air-dried and mounted on wooden supports with upright transverse surfaces. To accurately identify individual tree rings, the samples were polished with a razor blade and sandpaper (Smith et al. 2014). Sandpaper was only applied to samples with unclear boundaries to avoid generating heat, which could create volatile organic compounds (Balouet et al. 2007), and to minimize wood dust from accumulating in the vessel, which could complicate dendrochemical analysis (Locosselli et al. 2019). Tree ring width (TRW) was measured with a precision of 1 μm using a stereomicroscope, a Lintab 6 measuring table, and Tsap-Win software. Samples from the same tree were visually cross-dated to identify missing or false rings and subsequently averaged. The TRW series was cross-dated using Tsap-Win, and the accuracy was validated with COFECHA. (Holmes 1983).

### Sample selection and preparation for ED-XRF analysis

A total of 22 samples (one radius per tree) were selected for dendrochemical analysis, covering age ranges from 1945–2021 and 1998–2021. Nineteen were collected from the mining area: Mormorgruvan (8 samples), Haggruvan (3 samples), Varpgruvan (5 samples), and the drainage ditch (3 samples). Three additional samples were obtained from the reference site. Samples exhibiting decay, indistinct ring boundaries, or missing/false rings were excluded. The final selection also ensured proper radial orientation, as eccentric growth or an oblique boring angle could compromise the accuracy of ED-XRF measurements (Smith et al. 2014). Since mining activities ceased in the early twentieth century, including the closure of the Åtvidaberg copper smelter in 1902, no atmospheric emissions were expected during the sampled trees’ lifespans. Root uptake was therefore assumed to be the primary pathway for metal accumulation. To reflect this, trees were selected near waste rock piles and runoff ditches to maximize soil-related exposure (Watmough 1999). Thin wooden laths (1.2 mm) were cut using a twin-bladed circular saw and analyzed for elemental composition with the ITRAX Multiscanner (Cox Analytical Systems, www.coxsys.se) at the Tree-Ring Laboratory, Stockholm University. Chemical analysis was conducted using a Cr tube at 30 kV and 50 mA, with an exposure time of 3 s and a line scan step of 50 µm. The multiscanner produced elemental profiles that included light elements (e.g., aluminum and magnesium), heavy elements (e.g., lead), and radiographic images; this study focuses on Cu, Ni, and Zn.

To link the chemical profiles to the tree rings, WinDendro (Régent instruments Canada Inc. version 2012a) was used to extract pixel-based TRW data from the radiographic images. To correct for wood matrix effects, element counts were divided by the coherent scattering (Garivait et al. 1997; Smith et al. 2008). The average value for each element was then calculated per annual ring, excluding the first and last year to avoid edge and pith bias (Scharnweber et al. 2016).

### Soil sample collection and preparation

Soil samples were collected from beneath the sampled trees at a depth of approximately 20 cm. Due to the high waste rock content and limited soil near the waste rock piles, only 18 soil samples could be collected at varying distances from the trees. For trees growing along the drainage ditch, samples were taken 100 m upstream to obtain mineral soil, as these trees were rooted in peat. Samples were labeled according to the tree sample ID system, with an “S” added (e.g., MgS for Mormorgruvan). All samples were air-dried, homogenized using a ceramic mortar and pestle, compacted, covered with plastic film, and analyzed for Cu, Ni, and Zn using portable X-ray fluorescence (pXRF) with an Rh tube in soil mode for 60 s (Knight et al. 2021). For pH analysis, 5 g of homogenized soil was placed in 50 mL plastic tubes and 25 mL of deionized water. The mixture was shaken and allowed to settle for ~ 24 h (Nilsson et al. 2015). pH was measured using a Metrohm metal 691 pH Meter calibrated for a pH range of 4 to 7.

### Climate data

Climate data for 1998–2021 was sourced from the Swedish Meteorological and Hydrological Institute (SMHI n.d.-a). Daily temperature and precipitation data for the years up to 2001 were obtained from the Linköping-Malmslätt climate station (58°23′53′′N, 15°31′23′′E), located approximately 30 km north-northwest of the study site. From 2002 to 2021, data were sourced from the Gustorp D climate station (58°25′32"N, 16°8′28"E), situated about 30 km north-northeast of the study site. In 2009, this station was relocated 500 m west to (58°25′28′′N, 16°7′52′′E). The data were used to calculate the annual average temperature and total annual precipitation. Additionally, average temperatures and total precipitation for the season were calculated: Spring (March to May), Summer (June to August), Fall (September to November), Winter (December to February), and the growing season (April to September).

### Statistical approach

The Shapiro–Wilk test indicated a non-normal distribution of soil and dendrochemical data (Shapiro and Wilk 1965), promoting the use of the Mann–Whitney *U* test. This test is appropriate for small sample sizes, which is relevant given the limited number of reference samples (Nachar 2008). It was used to evaluate differences in Cu, Ni, and Zn levels in soil and tree samples and pH between the contaminated and reference sites. Spearman rank correlation tests were applied to assess relationships between soil heavy metal concentrations, pH, and tree-ring metal concentrations. Only soil samples collected near the corresponding tree samples were included; samples from the ditch were excluded due to a lack of direct spatial association. The median of aggregated values from the longest common period (1998–2021) was used to account for the temporal mismatch between soil samples and tree rings. This approach reduces variability and the influence of outliers, enabling a more robust comparison between sites. Spearman rank correlations were also performed to explore potential monotonic relationships between the full-length tree-ring elemental profiles and weather variables. Additionally, pairwise Spearman correlations between Cu, Zn, and Ni concentrations in tree rings were performed for each tree. The significance threshold (*p* < 0.05) was adjusted using the Bonferroni correction to mitigate Type I errors from multiple comparisons. While this conservative approach reduces false positives, it may increase the risk of Type II errors (VanderWeele and Mathur 2019). Tree-ring elemental data were normalized using Z-score transformation to facilitate comparison and detect anomalous years, defined as deviations exceeding 30% from the time series average (Balouet et al. 2012; Rocha et al. 2020).

## Results

### Soil heavy metal concentration and pH

**Fig. 2:**
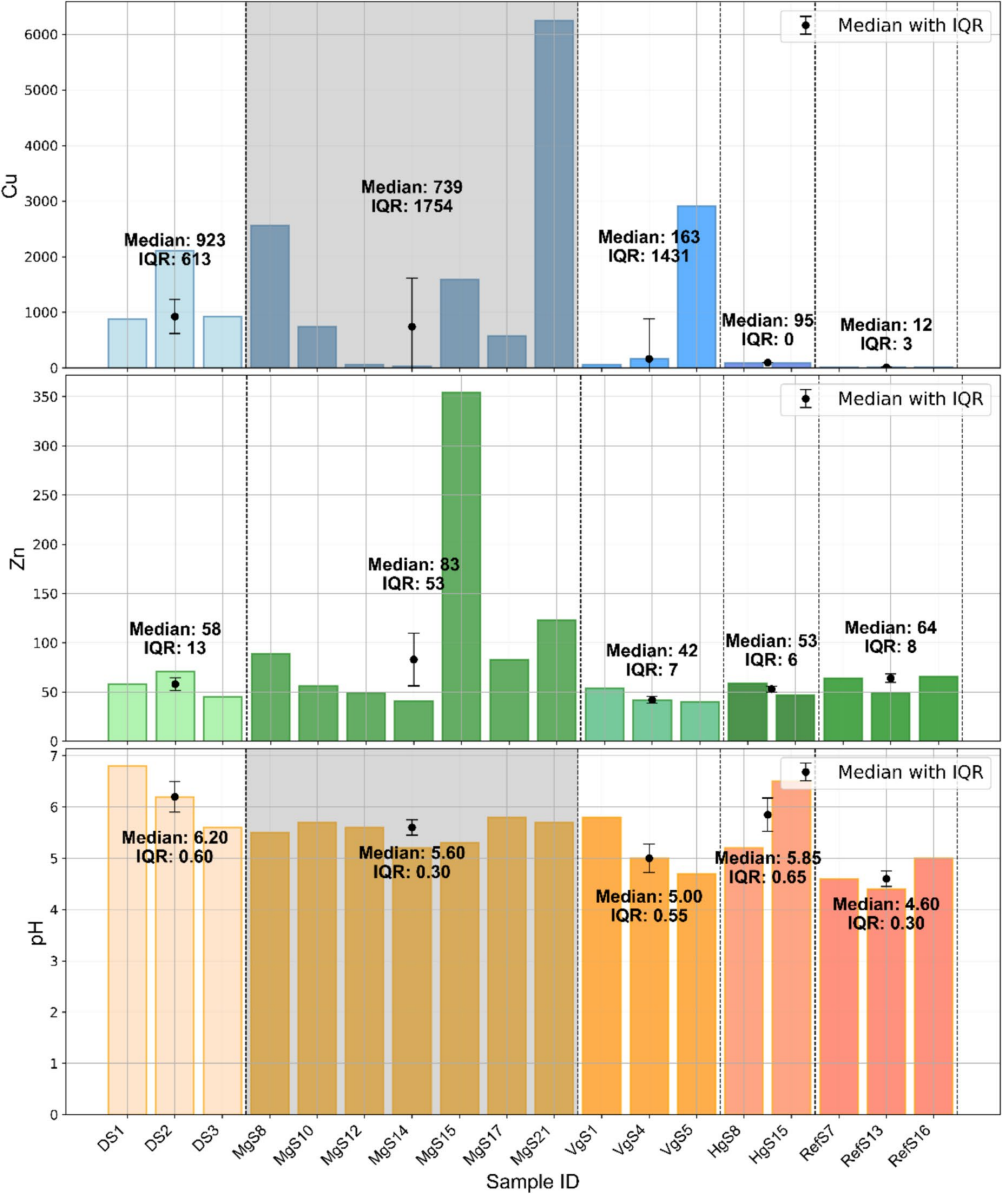
The median concentrations of Cu (top) and Zn (middle) (ppm) and the pH levels (bottom) in soil samples across five groups: Group 1 DS (Drainage ditch) (*n* = 3), Group 2 MgS (Mormorgruvan) (*n* = 7), Group 3 VgS (Varpgruvan) (*n* = 3), Group 4 HgS (Haggruvan) (*n* = 2), and Group 5 RefS (Reference site) (*n* = 3). The error bars represent the interquartile range (IQR), and vertical dashed lines separate the groups. Statistically significant differences from the reference group are shaded with Bonferroni corrected *p* value = 0.008 for Cu and *p* value = 0.011 for pH. Each bar represents the concentration and pH for each sample

Significantly elevated concentrations of Cu in the soil were observed at Mormorgruvan compared to the reference site (*p* = 0.008). While Cu concentrations at other contaminated sites did not reach statistical significance, their median values were markedly higher, ranging from 8 to 77 times above the reference median. Individual samples showed extreme Cu levels, with some concentrations up to 520 times higher than the reference (Fig. 2). There was substantial variability in Cu concentrations across all locations, except at Haggruvan, which exhibited more consistent values. In contrast, Zn concentrations did not differ significantly between sites. Interestingly, the reference site had a higher median Zn level than all contaminated sites, except for Mormorgruvan. At Mormorgruvan, sample MgS15 was a notable outlier, with a Zn concentration of 354 ppm. Nickel concentrations above the detection limit were found only in samples from the drainage ditch and sample VgS5, with values ranging between 27 and 161 ppm (data not shown). Soil pH was significantly higher at Mormorgruvan (pH = 5.60) compared to the reference site (pH = 4.60), showing an increase of one full pH unit (Fig. 2). Although elevated pH values and greater variability were also recorded at the other contaminated sites, these differences were not statistically significant. All samples—except for VgS5—had higher pH values, ranging from 4.70 to 6.80, than the reference site.

### Soil -tree-ring relationship

**Fig. 3:**
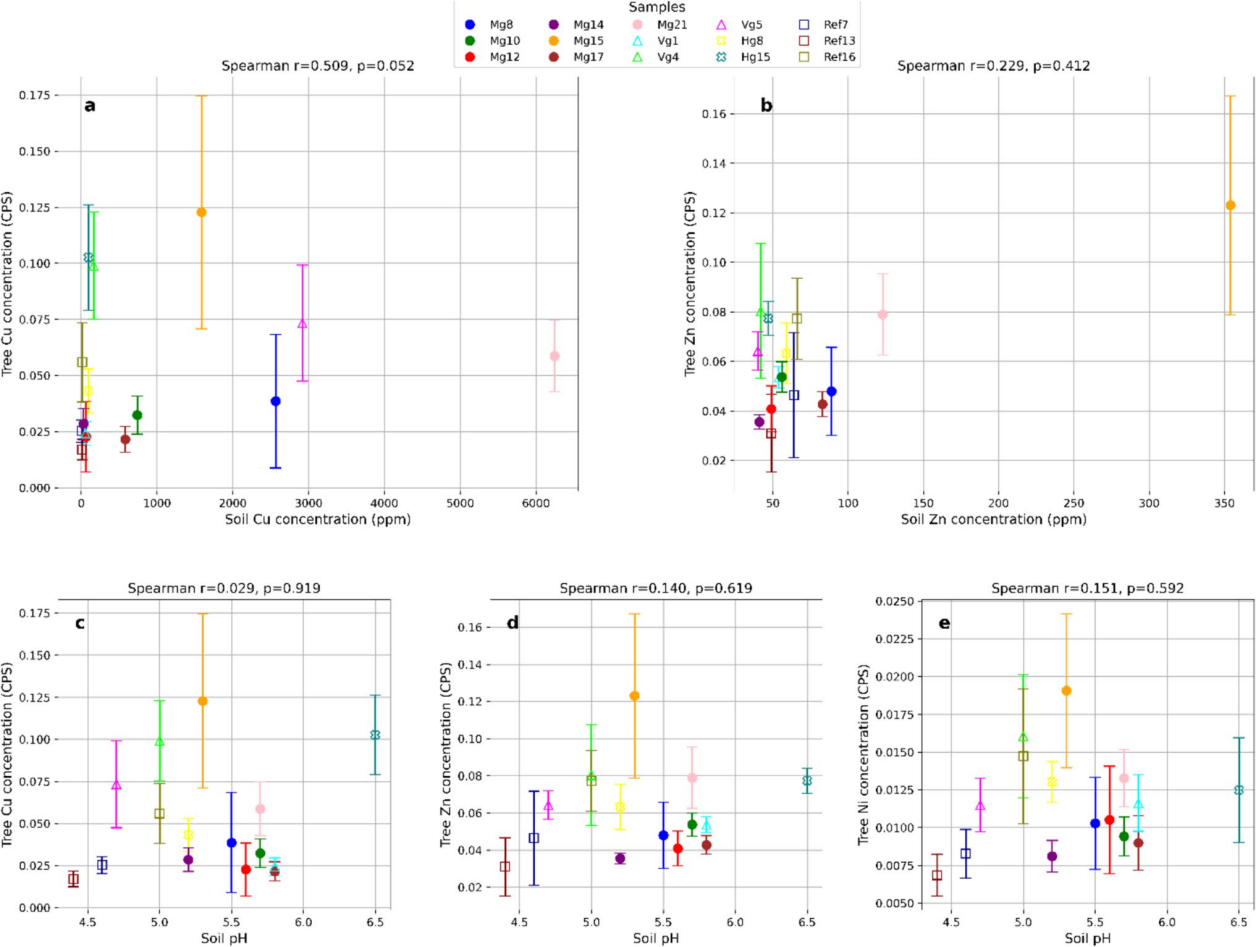
Results from the Spearman correlation test (p < 0.05) between soil and tree-ring concentrations observed for 1998-2021. Median values for tree-ring concentrations are visualized with error bars representing the IQR. Soil concentrations (measured in ppm or pH) are plotted on the x-axis, while accumulated tree-ring concentrations (measured in CPS, Counts Per Second) are on the y-axis. Relationships between soil and tree-ring metal concentrations and soil pH. (a) Soil Cu vs. tree Cu, (b) soil Zn vs. tree Zn, (c) soil pH vs. tree Cu, (d) soil pH vs. tree Zn, (e) soil pH vs. tree Ni. Symbols indicate samples: Mormorgruvan = filled circles, Haggruvan = crosses, Varpgruvan = triangles, and Reference site = squares

No significant correlations were found between soil concentrations of Cu or Zn and their respective concentrations in tree rings (*p* > 0.05; Fig. 3). The concentrations of these metals in tree rings showed considerable variability, often deviating from the site medians, which made it challenging to identify potential accumulation patterns. Similarly, the concentrations of Cu, Zn, and Ni in tree rings did not significantly correlate with soil pH. When examining cumulative metal concentrations in tree rings over the common period (1998–2021), 10 out of 19 trees exhibited significantly elevated levels of Cu and Ni, and 8 trees showed elevated Zn concentrations, relative to the reference site (Fig. 3). Trees with elevated concentrations of one metal frequently exhibited elevated levels of the other two metals as well (Fig. 6).

### Relationship between weather and tree rings

**Fig. 4:**
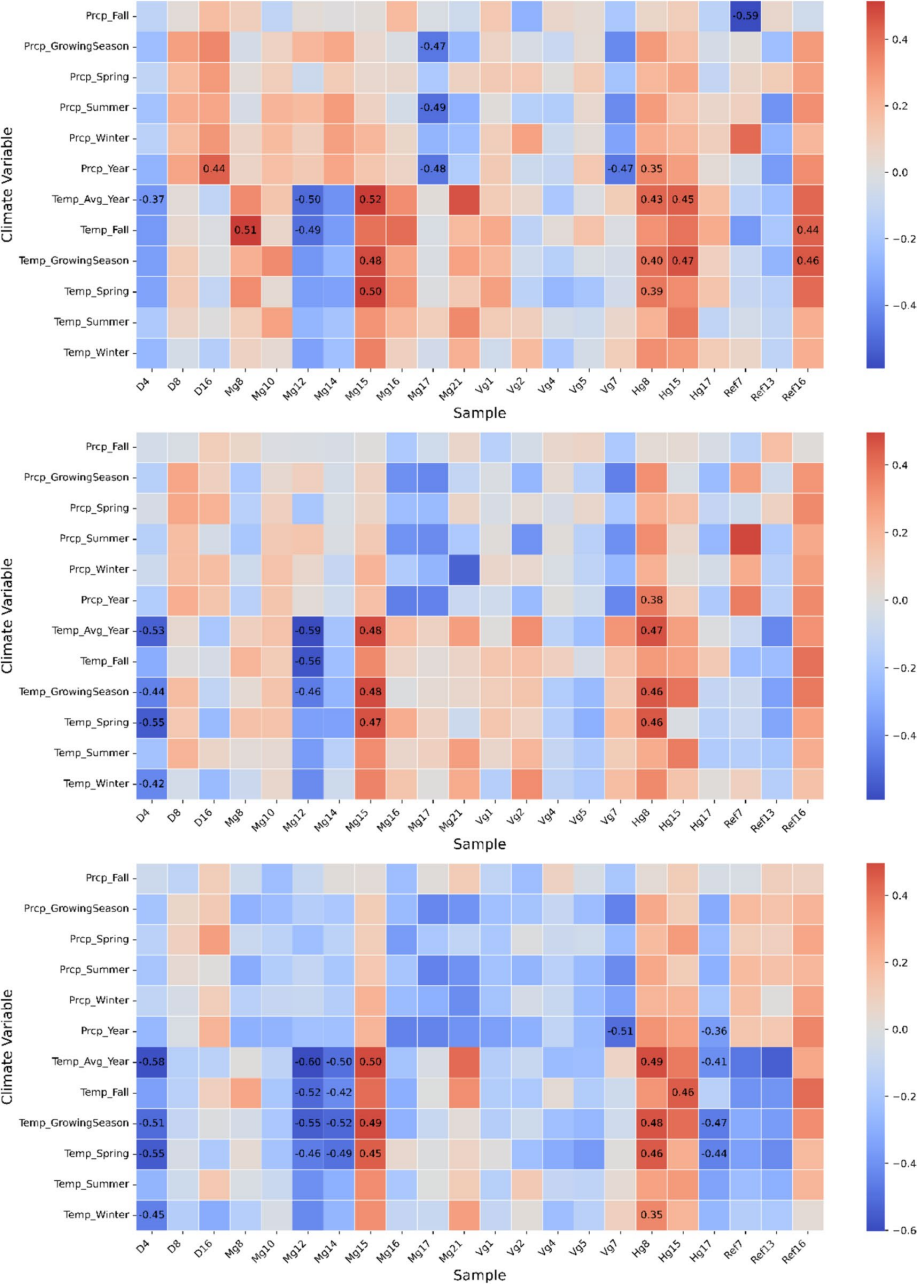
Correlation matrices between individual tree samples and climate variables across the entire length of each sample. Cu (upper), Ni (middle), and Zn (lower). The strength and direction of the correlation are represented by color, while the correlation coefficients are explicitly stated for significant correlations when the Bonferroni correction is applied (p<0.002)

Significant correlations were observed among all the elements studied, primarily related to temperature. A few exceptions were linked to precipitation variables (Fig. 4). Significant correlations were detected in samples from all contaminated sites except Varpgruvan. Cu generally showed positive relationships with temperature, whereas Zn tended to exhibit negative correlations. Ni showed the fewest significant associations. In general, samples that correlated with one climate variable also tended to show correlations with several others, particularly annual mean and growing season temperatures. In contrast, summer temperature was not significantly correlated with any of the samples. Spearman’s rank correlations were also calculated between annual tree-ring concentrations of Cu, Zn, and Ni for each tree (*n* = 22) over the full-time series. After Bonferroni correction for multiple testing, Cu–Zn correlations were significant in 14 trees, while Cu–Ni and Zn–Ni correlations were significant in 19 trees each (*p* = 0.00076). The average Spearman correlation coefficients were 0.59 for Cu–Zn and 0.72 for Cu–Ni and Zn–Ni.

### Dendrochemical profiles and anomalies

**Fig. 5:**
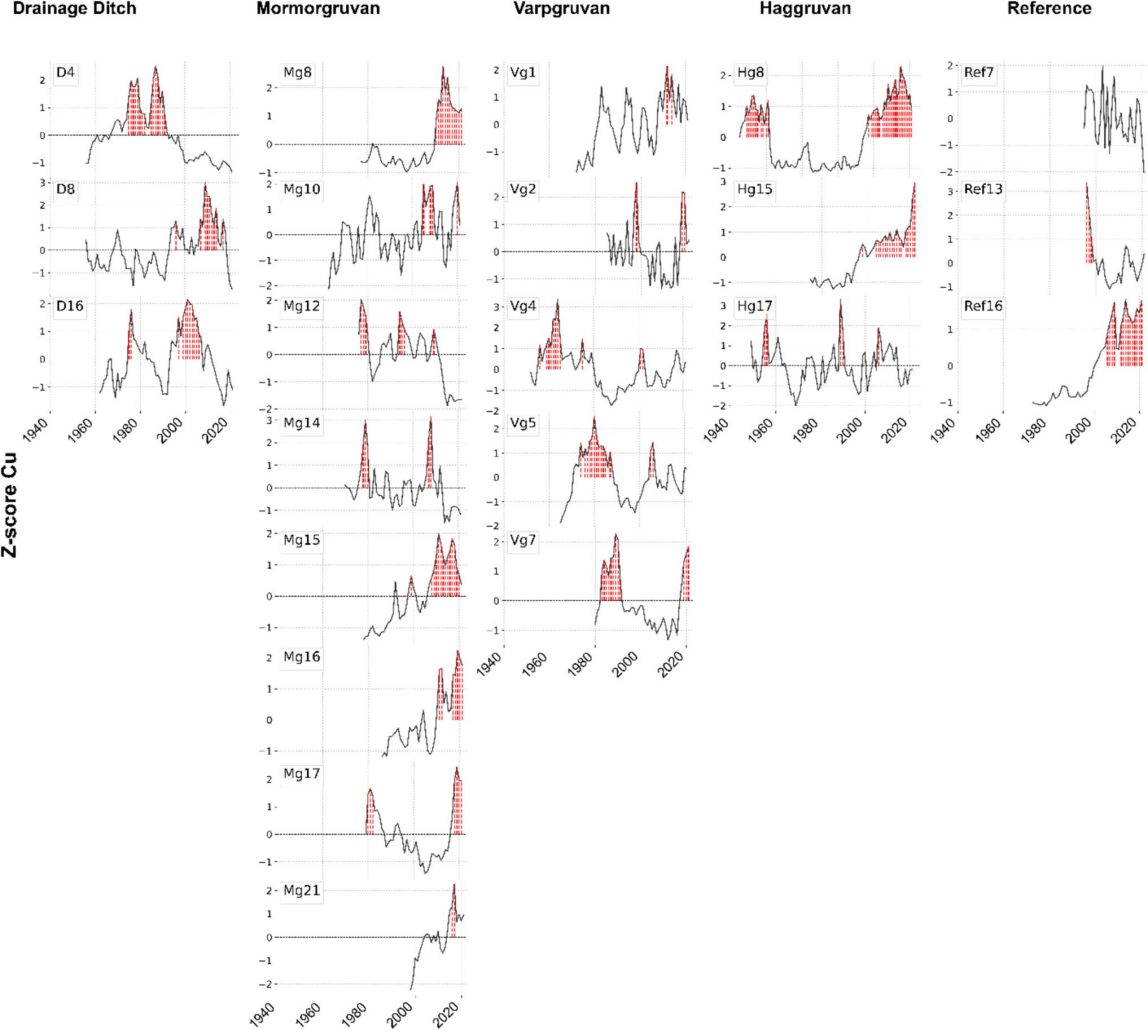
Tree-ring elemental profiles for Cu. Each column of subplots represents the different sampling sites (Drainage ditch Mormorgruvan, Varpgruvan, Haggruvan, and the Reference site, from left to right). The black line shows the normalized data (z-score). Red dashed vertical lines indicate anomalous years

**Fig. 6:**
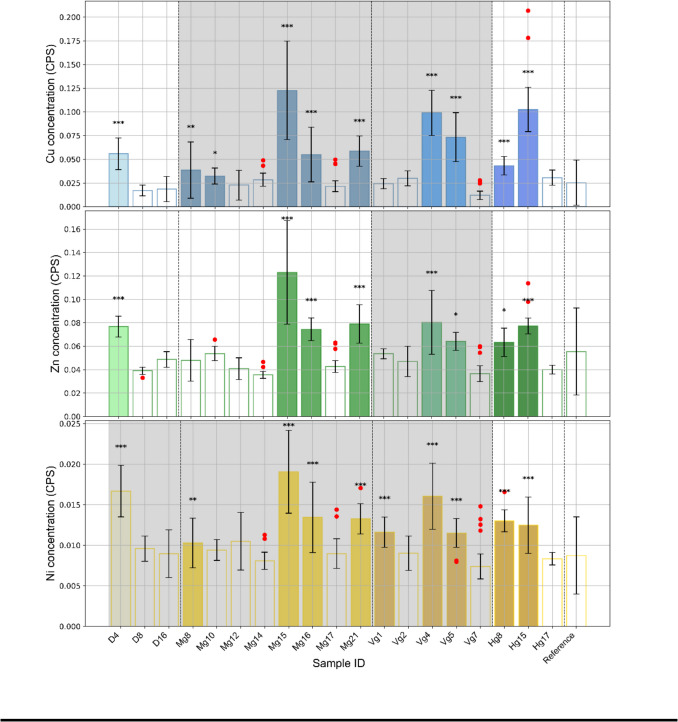
Median concentrations of Cu (top), Zn (middle), and Ni (bottom) for the longest common period (1998-2021) for individual samples. Samples from the reference site are grouped together. Error bars represent the interquartile range (IQR), and outliers are marked with red dots. Vertical dashed lines separate different groups of samples by location. Shaded areas indicate tree ring concentrations significantly different from the reference site at a significance level of p<0.0125 (Bonferroni correction applied) by the different sampling sites. Colored bars indicate individual samples with significant differences from the grouped reference site. Asterisks (*) above the error bars denote the level of significance: * = p<0.05, ** = p<0.01, *** = p<0.0026 (Bonferroni correction)

**Fig. 7:**
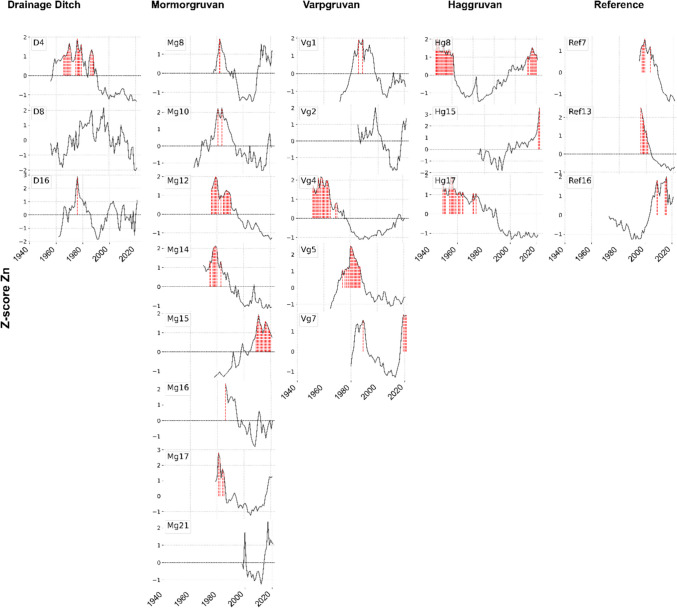
Tree-ring elemental profiles for Zn from individual samples. Each column of subplots represents the different sampling sites (Drainage ditch Mormorgruvan, Varpgruvan, Haggruvan, and the Reference site, from left to right). The black line shows the normalized data. Red dashed vertical lines indicate anomalous years, defined as values exceeding 30% of the time series mean concentration

**Fig. 8:**
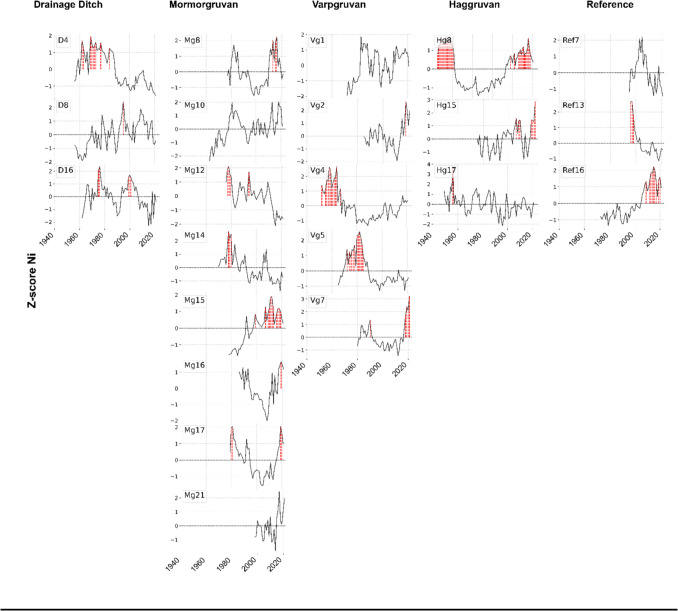
Tree-ring elemental profiles for Ni from individual samples. Each column of subplots represents the different sampling sites (Drainage ditch Mormorgruvan, Varpgruvan, Haggruvan, and the Reference site, from left to right). The black line shows the normalized data. Red dashed vertical lines indicate anomalous years, defined as values exceeding 30% of the time series mean concentration

**Fig. 9:**
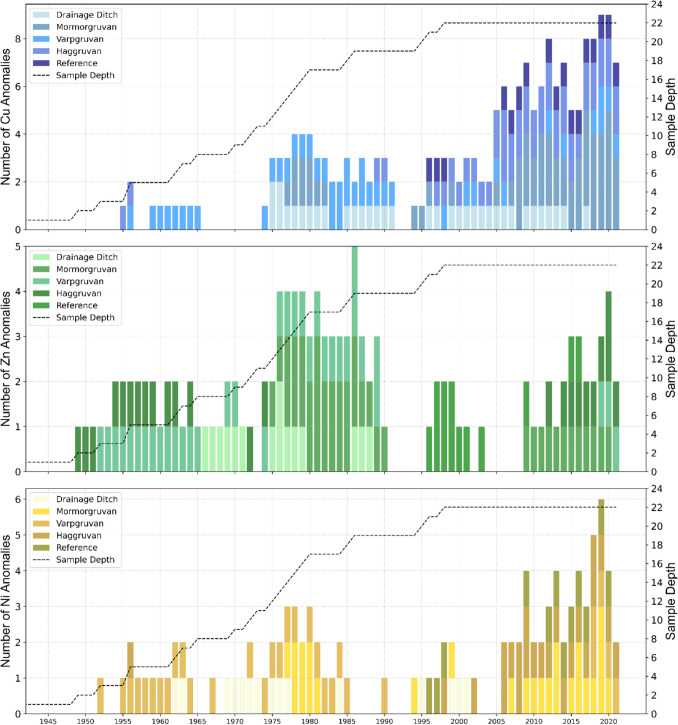
The annual distribution of anomalies for Cu (top), Zn (middle), and Ni (bottom) across all tree-ring samples. The bars represent the number of anomalies observed in all samples each year. The dashed line indicates sample depth. The x-axis shows the years, and the y-axis displays the number of time series exhibiting anomalies annually, highlighting periods of increased or decreased synchronicity in anomalies across the dataset

The dendrochemical profiles exhibit distinct and individual patterns (Fig. 5, Fig. 7, and Fig. 8), with low similarities between samples (mean correlation values: R̅ (Cu) = 0.0039, R̅ (Zn) = 0.0418, R̅ (Ni) = − 0.0256). These values indicate low agreement across the elemental profiles, reflecting minimal inter-tree correlation. Cu has the highest frequency of anomalies among the elements, often occurring in consecutive years. Comparable patterns of consecutive anomalies are also observed for Zn and Ni, although with less consistency. Notably, anomalies are not limited to contaminated sites but also appear in reference samples. Sample Ref 16 exhibits consecutive years of anomalies for Cu and Ni during the 2000 s, whereas Ref. 7 shows no anomalies for either element. A notable temporal trend in anomaly frequency is apparent despite variability among profiles. No anomalies were recorded for any element across all samples in 1992 and 1993. Following these years, anomalies increased steadily, peaking between 2015 and 2021. The highest frequencies were observed for Cu in 2019–2020 (9 anomalies), for Zn in 2020 (4 anomalies), in addition to a peak recorded during the 1980 s, and for Ni in 2019 (6 anomalies), (Fig. 9).

## Discussion

### Heavy metal and pH in soil

The results of the Mann–Whitney U-test for grouped soil samples should be interpreted with caution, due to the limited statistical power resulting from high variability and small sample sizes (Zimmerman 1987). Compared to the reference site, the marked variability and extreme Cu concentrations observed (Fig. 2) reflect the heterogeneity of contamination patterns associated with legacy mining activities (Austruy et al. 2019). While previous studies identified Zn and Ni as secondary pollutants (Wrafter 2004), their lower concentrations in this study may reflect reduced mobility due to natural attenuation processes in older mining areas (Anawar 2013), such as the depletion of reactive sulfide minerals. Ni was predominantly detected in the ditch, likely due to runoff accumulation from multiple mines. In contrast, Zn’s higher mobility may cause it to leach deeper into the soil profile, reducing its presence in surface samples (Kabata-Pendias and Szteke 2015).

The acidic conditions at the reference site align with typical pH values for coniferous forest soils (Augusto et al. 2002). By contrast, the elevated pH levels at contaminated sites are unexpected, given the high sulfide content typically associated with AMD (Simate and Ndlovu 2014). Seasonal fluctuations in pH (Sandén 1991) may temporarily raise pH levels, while natural attenuation processes could suppress ongoing acid generation over time (Anawar 2013). This complexity highlights the dynamic nature of soil geochemistry in post-mining landscapes and emphasizes the importance of using multiple samples to characterize the chemical environment surrounding tree roots accurately.

### The relationship between soil and tree rings

The lack of a consistent relationship between Cu or Zn concentrations in soil and their accumulation in tree rings (Fig. 3) suggests that soil metal concentration alone cannot reliably predict bioaccumulation, as previously noted by Greger (1999) and Kabata-Pendias (2004). Tree sample Mg21 exemplifies this, with the corresponding soil sample showing Cu levels over 500 times higher than at the reference site (Fig. 2). Yet, the median Cu concentration in the tree rings is similar to that of Ref13 (Fig. 3). This discrepancy likely reflects a combination of temporal and spatial mismatches that decouple momentary soil conditions from the multi-year accumulation signal preserved in tree rings. While median tree-ring concentrations provide a robust summary of uptake over a 24-year period, seasonal and interannual variability in soil pH and metal mobility (Sandén 1991) means that a single soil sample represents only a snapshot in time. Such variability may explain the absence of significant correlations between pH and tree-ring metal concentrations, despite pH being a well-established regulator of metal bioavailability (Antoniadis et al. 2017). Moreover, excluding the outermost ring during ED-XRF analysis (Scharnweber et al. 2016) reinforces this temporal disconnect by preventing year-specific soil and tree-ring data comparison.

It is also uncertain whether the soil samples accurately represent the rooting environment. Post-mining landscapes are inherently heterogeneous, both physically and chemically (Festin et al. 2019). In such settings, the extensive root systems of aspen (Myking et al. 2011) interact with a fine-scale mosaic of microsites shaped by waste rock, runoff channels, and variation in organic matter, pH, and microbial activity. These conditions may contribute to inter- and intra-tree variability (Watmough and Hutchinson 2003). However, spatial variability alone cannot fully explain the observed uptake patterns; chemical processes governing long-term bioavailability must also be considered. Metals’ chemical form and stability are critical, as aging processes gradually immobilize metals by binding them to soil constituents such as clay particles, organic matter, and Fe/Mn oxides (Lock and Janssen. 2003). Since mining ceased over a century ago, much of the residual metal pool is likely in less mobile forms. Thus, despite high Cu concentrations in soil, long-term immobilization may reduce the bioavailable fraction, limiting uptake. Conversely, the lower pH at the reference site may enhance solubility, resulting in comparable or even higher uptake than in contaminated soils. This may account for the lack of elevated accumulation observed in nearly half of the trees growing on contaminated soils.

Trees with significantly increased uptake may have roots accessing microsites where environmental fluctuations, such as changes in pH, redox potential, or physical disturbances, can remobilize otherwise stable metal pools (Nowack et al. 2010), increasing local bioavailability. The frequent co-accumulation of Cu, Ni, and Zn likely reflects simultaneous mobilization of multiple elements under such conditions, in combination with broad-specificity uptake systems that facilitate absorption via shared non-selective transporters (Yruela 2009). The lack of correlation likely reflects methodological limitations and spatial heterogeneity, while uptake differences may arise from long-term immobilization and localized remobilization processes. To accurately evaluate how dendrochemical signals reflect soil chemistry, temporally and spatially resolved soil sampling is required.

### Temperature, precipitation, and tree ring accumulation

Precipitation is crucial in leaching and transporting heavy metals (Sandén 1991). Therefore, the absence of significant correlations between rainfall and dendrochemical profiles was unexpected (Fig. 4). This discrepancy may partly reflect the distance between the weather station and the study site, as local precipitation can vary significantly. Additionally, centuries of mining have fractured the bedrock and increased hydrological conductivity, which, combined with minimal topsoil (Wrafter 2004), promotes rapid drainage and limits interactions within the root zone. In response, *Populus* species may exhibit root plasticity, enabling deeper rooting and access to more stable water sources (Rosso et al. 2023). This adaptation likely contributed to the lack of a precipitation signal.

Temperature had a more consistent influence on metal uptake (Fig. 4). Elevated temperatures can increase metal concentrations in the soil solution through enhanced microbial activity, solubility, and evapoconcentration (Punia 2021). However, limited soil moisture can also lower transpiration rates, constraining uptake. This physiological limitation during summer may explain the absence of significant correlations between metal accumulation and summer temperature in our data. In contrast, yearly and growing season temperature metrics include spring and autumn, when elevated temperatures enhance metal mobility, but higher soil moisture maintains transpiration rates, facilitating metal uptake. This suggests that temperature-induced increases in metal mobility, combined with plant physiological processes such as transpiration regulation and uptake efficiency (Rosso et al. 2023), rather than changes in rainfall, drive uptake patterns under climatic variability at this site.

These broader climatic and physiological processes may interact with element-specific properties, contributing to the variable response patterns observed for Cu, Zn, and Ni. We observed primarily positive correlations between Cu and temperature, whereas Zn primarily exhibited negative correlations. These patterns likely reflect differences in the physiological uptake mechanisms of these elements. Tripathi et al. (2018) reported increased Cu and reduced Zn levels in *Populus* clones under drought, indicating metal-specific regulation and transport pathways under environmental stress. Elevated Cu availability can further suppress Zn uptake both at the root-soil interface (Toselli et al. 2008; Yruela 2009) and during xylem loading due to competition for binding sites (Kutrowska et al. 2017; Österås et al. 2004). Although our data show predominantly moderate to strong positive correlations between tree-ring Cu and Zn concentrations, these were generally weaker and less consistently significant than those involving Cu–Ni and Zn–Ni. This outcome could indicate partial antagonistic interactions that modulate but do not preclude co-accumulation. However, the contrasting temperature responses of Cu and Zn are unlikely to result from antagonism alone. Other contributing factors, such as differences in solubility, speciation, or root uptake kinetics under varying climatic conditions, are likely to influence uptake behavior. Consequently, temperature-driven accumulation of these elements appears to arise from a combination of metal-specific physiological regulation, environmental availability, and potential inter-element interactions. Whereas Cu and Zn showed somewhat coherent uptake patterns with temperature, Ni exhibited more irregular behavior, lacking consistent correlations. This may reflect Ni’s distinct chemical properties and uptake pathways. Unlike Cu and Zn, Ni is a non-essential element less tightly regulated by plant physiology. Instead, soil chemistry largely governs its uptake, including pH and cation competition, where Cu and Zn may inhibit Ni’s bioavailability (Kabata-Pendias and Szteke. 2015).

Temperature thus exerted a more consistent influence on metal accumulation than precipitation, indicating that temperature-driven processes dominate uptake at this site. The differing responses among elements highlight the importance of considering metal-specific pathways and physiological regulation when interpreting climatic effects in dendrochemical records. Future work should investigate how shifts in water availability and plant function under climate change modulate metal uptake over time.

### Dendrochemical patterns and anomalies

The dendrochemical profiles reveal highly variable and distinct uptake patterns (Figs. 5, Fig. 7, and Fig. 8), characterized by low mean correlation values for Cu (R̅ = 0.0039), Zn (R̅ = 0.0418), and Ni (R̅ = − 0.0256). This individualistic behavior, with minimal agreement in heavy metal uptake among trees, suggests that localized environmental factors and site-specific conditions strongly modulate annual accumulation. These patterns are consistent with previous studies (Locosselli et al. 2019; Odabasi et al. 2016; Rocha et al. 2020), which also highlight the influence of local environmental variability and external pollution sources on dendrochemical records. Despite this inter-tree variability, the timing of anomalies exhibits shared temporal trends (Fig. 9). Multi-year anomalies for Cu, Zn, and Ni—some extending over 10 to 20 years (e.g., Mg8, Mg15, Hg8)—suggest persistent environmental drivers of anomalous uptake. Peaks in these trends likely reflect periods of enhanced metal bioavailability. However, interpretation is complicated by xylem properties. Radial translocation of metals across ring boundaries is well-documented (Binda et al. 2021; Lepp 1975; Watmough 1999). Metals translocate in symplast and apoplast within the sapwood, the physiologically active part of the stem responsible for water transport (Smith and Shortle 1996). In *Populus tremula*, a diffuse-porous species, boundaries between heartwood and sapwood are often indistinct, with a broad sapwood zone. Water transport is most active in the outer, younger rings and gradually decreases toward the stem center (Lambs and Muller 2002). This anatomical characteristic enables extended radial mobility of elements, reducing temporal resolution and introducing uncertainty when attributing uptake events to specific calendar years (Scharnweber et al. 2023). Consequently, dendrochemical anomalies must be interpreted cautiously, especially for moderately mobile elements such as Cu and Zn, whose mobility may obscure the temporal resolution of dendroforensic records. In contrast, the lower mobility of Ni likely preserves more precise temporal signals (Cutter and Guyette 1993).

Despite these complexities, the observed anomalies demonstrate a collective temporal coherence. Notably, no anomalies were detected for Cu, Zn, or Ni in 1992 or 1993 (Fig. 9), which coincided with the most severe summer drought recorded in a century (SMHI n.d.-b). The reduced water availability during this period likely hindered metal dissolution and transport into the root zone, resulting in reduced uptake and an absence of detectable anomalies. Following this drought, the frequency of anomalies increased, aligning with a period of rising temperatures and increased precipitation (SMHI n.d.-a). As previously discussed, elevated temperatures enhance metal bioavailability (Punia 2021) while increased precipitation helps maintain sufficient water availability in the root zone, thereby supporting heavy metal uptake. These climatic factors likely amplified the observed trends in anomalous heavy metal accumulation. Anthropogenic disturbances, such as the reshaping of waste rock piles or the construction of drainage ditches at the study site (Wrafter 2004), may have further mobilized metals; however, the timing and extent of these activities remain unclear.

Copper exhibited the highest number of anomalies, potentially due to its moderate mobility within xylem, antagonistic interactions with other ions, or its persistence in biologically available forms under fluctuating environmental conditions (Furini 2012). This underscores the importance of element-specific properties, including mobility, reactivity, and physiological regulation, in mediating tree-ring metal accumulation under variable environmental conditions. The low R̅ values highlight spatial variability in heavy metal uptake, driven by localized factors such as soil composition, pH, and hydrology. These results emphasize the need for integrated spatial and temporal analyses in dendrochemical studies to disentangle the complex interplay between environmental conditions and biological factors.

### Methodological reflections and future directions

Many dendroforensic studies analyze tree rings from years predating contamination to establish baseline elemental profiles and distinguish natural variation from pollution signals (Balouet et al. 2007; Chen et al. 2021). However, in areas with long industrial histories, finding trees that predate contamination is often challenging (Rocha et al. 2020). This scenario is evident in this study, where mining activities ceased decades before the establishment of the sampled trees, thereby precluding a direct assessment of pre-contamination uptake patterns. Reference samples from an unpolluted site were used to provide context. While reference samples offer valuable insights, they do not account for site-specific factors such as soil composition, chemistry, or hydrology, which may influence dendrochemical signals independently of pollution. To overcome these limitations, a careful design sampling strategy incorporating historical and environmental characteristics is crucial for accurate interpretation of dendrochemical data.

#### Implications for sampling strategies

Dendrochemistry has been used to date pollution events since the 1970 s (Lepp 1975), yet standardized sampling protocols remain lacking. This study highlight the need for structured sampling strategies to improve the interpretation of contamination signals in tree rings. One significant limitation lies in the reliance on reference samples for pre-contamination comparisons. The observation of higher elemental concentrations and anomalies in the reference samples compared to the contaminated ones (Fig. 5, Fig. 7, Fig. 8, and Fig. 9) raises concerns about their reliability and underscore the importance of selecting trees that predate contamination whenever possible. Soil properties are another unresolved factor. The substantial variability observed in elemental profiles suggests that sampling trees from comparable soil types is essential for isolating the influence of soil characteristics on uptake (Binda et al. 2021). Targeted sampling within homogeneous soil environments may help clarify how geochemical conditions affect dendrochemical signals.

In addition to soil-related influences, the sampling strategy within trees themselves introduces another layer of complexity. Like many dendrochemical studies, this research relied on a single core per tree. Gavrikov et al. (2023), however, raised the critical question of *whether a single core can represent all the stem*? Their findings, supported by Canning et al. (2023), Garbe-Schönberg et al. (1997), and Martin et al. (2006), revealed significant elemental variability among cores taken from the same tree. This variability can stem from anatomical differences in wood structure, differential root exposure to contaminants, and localized environmental factors influencing uptake and transport. While this study focused on variability between trees rather than within stems, the low agreement observed may partly reflect limitations of single-core sampling. Incorporating multiple cores per tree could reduce sampling bias and provide a more accurate representation of elemental distributions. Addressing within-stem variability through multi-core strategies is essential to improve the robustness and reliability of dendrochemical interpretations.

#### Environmental and multivariate considerations

The chemical profiles and anomalies observed in this study indicate that heavy metal uptake cannot be attributed to temperature or precipitation alone. Instead, they reflect a complex interaction between climate variability, soil properties, and plant physiological responses (Binda et al. 2021). Incorporating additional climate-related parameters such as relative humidity, evapotranspiration, soil moisture, and seasonal extremes into multivariate analyses could improve understanding of how changing climate regimes influence metal mobility, leaching, and root uptake in post-mining landscapes. Beyond climatic influences, interactions among elements further complicate dendrochemical interpretation. Antagonistic relationships between Cu and Zn in soil and the xylem may significantly affect uptake patterns. Expanding the analysis to include a broader range of elements could reveal key dynamics in metal accumulation. Although this study focused on Cu, Zn, and Ni, utilizing the full capacity of ED-XRF to assess a broader elemental spectrum offers an opportunity to explore inter-element relationships. Investigating elemental ratios may thus enhance our understanding of the physiological and environmental controls shaping heavy metal accumulation in tree rings.

## Conclusion

This study provides insights into the potential of tree-ring analysis as a tool in environmental forensics to assess historical pollution trends and understand ecosystem impacts. While no significant correlations were identified between soil heavy metal concentrations and tree-ring accumulation, the elevated uptake of Cu, Ni, and Zn indicates that factors beyond soil concentrations, such as climatic conditions or antagonistic interactions between soil and xylem elements, play crucial roles. Temperature exhibited a greater influence on heavy metal uptake, whereas precipitation appeared to have a more limited effect. Anomalous uptake was suppressed during severe drought but increased under warmer, wetter conditions, highlighting the necessity for a multivariate approach to understand climate-related impacts comprehensively. The varied chemical patterns observed among the sampled trees further emphasize the significance of localized conditions. To improve the reliability of dendroforensic methods, future research should incorporate multi-core analysis and account for environmental variability. These refinements would strengthen tree-ring analysis for long-term environmental monitoring and pollution assessment.

## Data Availability

The data will be made available on request.

## Appendix

Figs. 6, 7, 8 and 9
